# Editorial: The mechanism and novel strategies of overcoming resistance of hematological malignancies to CAR-T cell killing

**DOI:** 10.3389/fimmu.2022.1093339

**Published:** 2022-12-09

**Authors:** Tingting Zhang, Tiantian Yu, Xingcheng Yang, Weiwei Tian, Jia Wei

**Affiliations:** ^1^ Cancer center, Shanxi Bethune Hospital, Shanxi Academy of Medical Sciences, Tongji Shanxi Hospital, Third Hospital of Shanxi Medical University, Taiyuan, Shanxi, China; ^2^ Department of Hematology, Shanxi Bethune Hospital, Shanxi Academy of Medical Sciences, Tongji Shanxi Hospital, Third Hospital of Shanxi Medical University, Taiyuan, Shanxi, China; ^3^ Sino-German Joint Oncological Research Laboratory, Shanxi Bethune Hospital, Shanxi Academy of Medical Sciences, Taiyuan, Shanxi, China; ^4^ Division of Hematopathology, Duke University Medical Center and Duke Cancer Institute, Durham, NC, United States; ^5^ Department of Hematology, Tongji Hospital, Tongji Medical College, Huazhong University of Science and Technology, Wuhan, Hubei, China; ^6^ Immunotherapy Research Center for Hematologic Diseases of Hubei Province, Wuhan, Hubei, China

**Keywords:** CAR-T, hematological malignancies, mechanism, novel strategies, overcome resistance

## Mechanism of resistance

Our previous review (1) and Xiao et al. have summarized the key resistance mechanisms of CAR-T cell therapy in B‐cell hematological malignancies. According to the tumor antigen expression at relapse, CAR-T failure may be classified to CD19^-^ relapse and CD19^+^ relapse and the former is generally considered the most common mechanism. As for CD19^-^ relapse, the underlying biological processes includes CD19 genetic mutations, such as point mutations or loss of heterozygosity of CD19; posttranscriptional regulation, such as alternative splicing; epigenetic modifications, such as methylation silencing; antigen epitope masking due to CAR-T cell trogocytosis; lineage switching driven by oncogenic genes; and pre‐existing CD19^‐^ tumor clones’ expansion under selective pressure (1). As for CD19^+^ relapse, any potential factors that may cause poor quality and quantity of CAR-T cells might be attributed to relapse. The unfavorable factors include: 1) high baseline tumor burden or previous multi-line of chemotherapy, which may damage T cells leading to production failure or inadequate quality during CAR-T cell preparation; 2) signaling abnormalities that are associated with T-cell exhaustion, such as IL‐6 signaling pathway, phosphatidylinositol-3-kinase (PI3K) signaling pathway, and/or impaired death receptor signaling, which limit the *in vivo* persistence and potency of CAR-T cells; 3) sustained antigenic stimulation *in vivo* inducing CAR-T cell exhaustion through transcriptomic and epigenetic modulations; 4) most clinically accessible CAR-T cell products have a murine-derived single-chain variable segment, that triggers immunological rejection; 5) activation-induced cell death effect due to repeated antigen stimulation leading to the upregulation of death receptors and low persistence of T cells. The dysregulated immunosuppressive microenvironment consisting of suppressive immune cell populations, inhibitory immune checkpoint molecule, cytokines, and enzyme, also contribute to impaired cytotoxicity of CAR-T cells. Besides, tumor cells themselves resist CAR-T cell killing *via* intrinsic or extrinsic antiapoptotic pathways, like upregulating the anti-apoptotic proteins expression (BCL-2, BCL-XL or MCL-1) and downregulating the expression of death-ligand/receptor.

**Figure 1:**
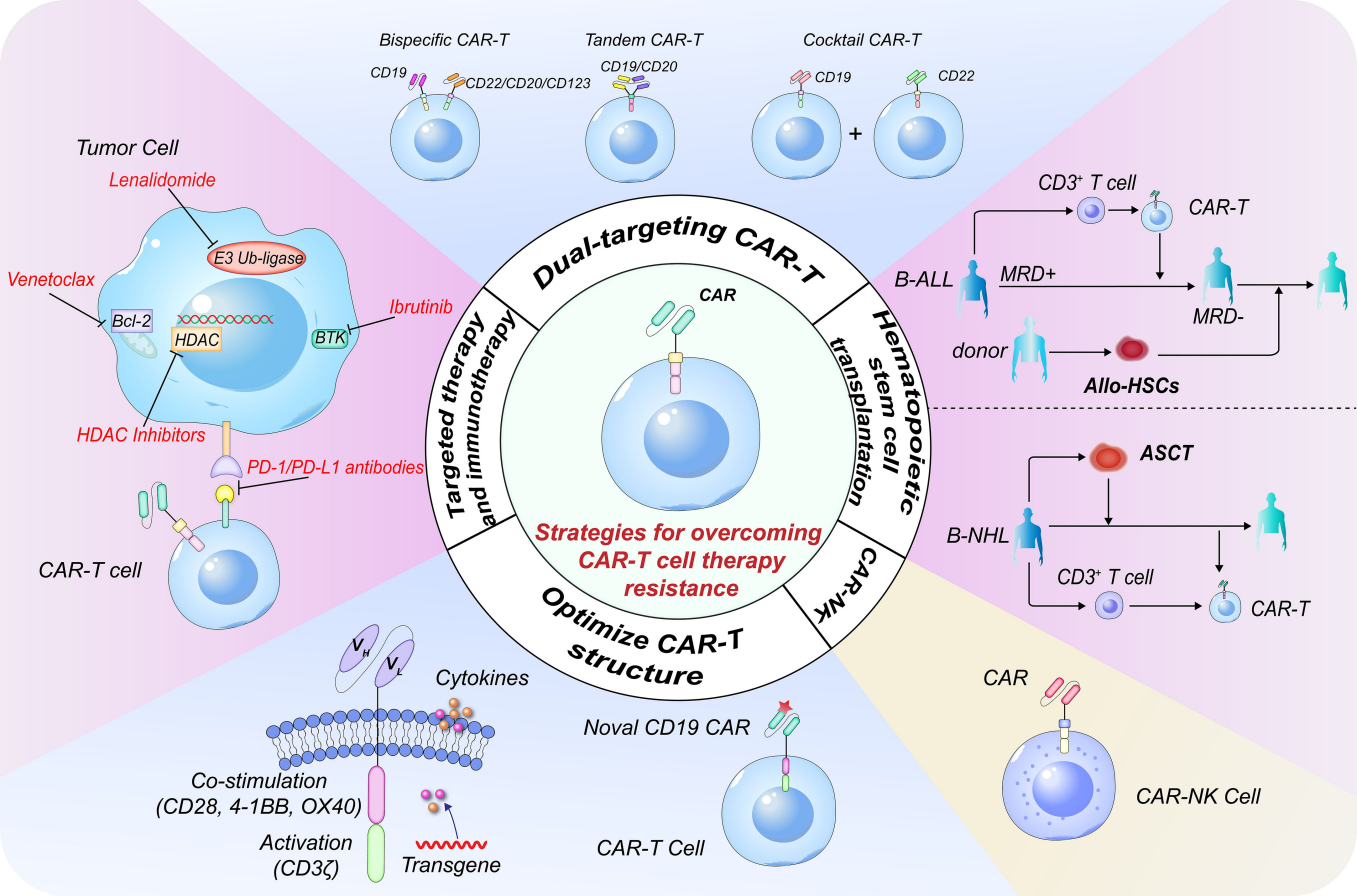
Strategies to overcome resistance in CD19 CAR-T cell therapy. Targeting multiple antigens is a pivotal strategy for overcoming CD19^-^ relapse. The three most common dual-targeting CAR-T cell therapeutic strategies include bispecific CAR-T cells, tandem CAR-T cells, and ‘cocktail’ CAR-T cell therapy. Constructing novel CD19 CAR-T cells targeting different binding epitopes is an alternative for CD19^-^ relapse. Constructing next-generation CAR-T cells with different co-stimulatory receptor domains or cytokines can further enhance the CAR-T cell persistence. Combination therapies with traditional therapeutic systems or targeted therapies are very important. The combination of CAR-T cells with HDACi, BTKi, BCL-2i or PD-1/PD-L1 antibody can restore the expression of tumor antigens, enhance *in vivo* persistence and efficacy of CAR-T cells and result in synergistic anti-tumor effects. CAR-T cell therapy can effectively eliminate MRD and is a powerful strategy to bridge allo-SCT in B-ALL. CAR-T cell therapy combined with ASCT has significant synergistic effects in B-NHL. CAR-NK cell is another promising approach for patients with difficulty or inaccessible to CAR-T therapy. CAR, chimeric antigen receptor; HDACi, histone deacetylase inhibitor; BTKi, Bruton’s tyrosine kinase inhibitor; BCL-2i, B-cell lymphoma-2 inhibitor; MRD, minimal residual disease; allo-SCT, allo-hematopoietic stem cell transplantation; B-ALL, B-cell acute lymphoblastic leukemia; ASCT, autologous stem cell transplantation; B-NHL, B-cell non-Hodgkin lymphoma.

In summary, the resistance mechanism can be mainly summarized into three aspects: (1) the CAR-T cells' diminished ability to work and short lifespan *in vivo*; (2) downregulation/loss of tumor surface antigen or tumor cell-intrinsic resistance to death signaling; and (3) the dysregulated immunosuppressive microenvironment. A complete understanding of CAR-T cell-centric, tumor-centric, and tumor microenvironment related obstacles would help unlock the potential of the legendary CAR-T cell therapy and provide rational strategies for combination therapies as well. Several strategies to overcome the causes of CAR-T cell resistance have been explored and proven to be successful in clinical practice (**Figure 1**).

## Novel CD19 CAR-T or dual CAR-T cells to overcome CD19^-^ relapse

Following CD19 targeted CAR-T cell therapy for B‐cell malignancies, around 50% patients experienced relapse due to CD19^-^ malignant clone expansion. Nan et al. reported a case with CD19^-^ relapse, who was unresponsive to FMC63 CAR-T cells. A p.163 R>L site mutation within exon 3 of CD19 locus was found in tumor biopsy of the progression site of the patient. Then, a novel CD19 CAR-T cell product derived from clone 21D4 was constructed, which binds to a different epitope. Sustained complete remission (CR) was achieved post this novel CD19 CAR-T cells’ infusion. Another investigation demonstrated that 21D4 CAR-T cells exhibited potent antitumor effect on CD19 exon 1 and 2 mutant B-cell lymphomas (2). Moreover, CAR T‐cells adding costimulatory domains (e.g., 4‐1BB and/or CD40) or inducing immunostimulatory cytokines secretion (e.g., IL-2 and/or IL-15) can largely boost CAR T‐cell function and persistence.

Dual-targeting CAR-T cell therapy is also a feasible treatment strategy for CD19^-^ relapse. The three most popular dual-targeting methodologies at the moment are tandem CAR-T cells, which involve a single CAR molecule with two antigen-binding domains in a single CAR-T cell, bispecific CAR-T cells, which contain two isolated CARs with one antigen-binding domain each in a single CAR-T cell, and sequential infusion of two single-targeted CAR-T cells. In our previous study, 66 patients with relapsed/refractory (r/r) B-cell non-Hodgkin lymphoma (B-NHL) were recruited and treated with CAR19/22 cocktail T-cell sequential administration, of which 87.7% patients achieved objective response, including 47.7% with CR, with a long median progression-free survival (PFS) of 14.8 months (3). Besides, this cocktail therapy resulted in much higher minimal residual disease (MRD)-negative response rate and reduced the risk of antigen-escape recurrence, such that only one patient experienced antigen-loss relapse during follow-up in another larger r/r B-cell malignancies sample cohort (4). Cordoba et al. enrolled 15 subjects with r/r B cell acute lymphoblastic leukemia (B-ALL) who supplemented with bispecific CAR-T cells expressing both anti-CD19 and anti-CD22 CARs, of which 86% patients achieved CR, with 60% and 32% of overall and event-free survival rates at one year (5). Tong et al. recruited 28 r/r NHL cases who experienced tandem CAR-T cells therapy targeting CD19/CD20. The CR and PFS rates at 12 months were 71% and 64%, respectively (6). Dual-targeting CAR-T cell treatment has the probability of accomplishing long-term disease control and prolonging the survival of subjects.

## Incorporating CAR-T cell therapy into traditional therapy system

Xiao et al. provided a comprehensive description of the promising strategies to further enhance the antitumor activity and overcome resistance of CAR-T cell therapy, including incorporating it into the traditional therapeutic system, such as hematopoietic stem cell transplantation (HSCT), chemotherapy and radiotherapy.

HSCT has been considered as the only possibly curative strategy and pursued as a potential manner to optimize the CAR-T-cell potency. Xiao et al. summarized four trial regimens and reviewed selective trials using the combinations of CAR-T with HSCT. In individuals with high tumor burden and poor prognostic factors, early allogeneic HSCT (allo-HSCT, within 80 days) is more effective at preventing cancer relapse. Analysis conducted by Hu et al. revealed that consolidative CAR-T therapy effectively eliminated pre-HSCT MRD, combined with HSCT significantly improved 3-year overall survival and 3-year leukemia-free survival compared to the chemotherapy group in pediatric patients with the first relapse of B-ALL.

Autologous HSCT (ASCT) is a standard consolidation or salvage therapy for most B-NHL cases. We incorporated CAR19/22 T-cell cocktail therapy into ASCT, which showed superior overall response rate and long-term survival in r/r B-NHL, including TP53-disrupted patients (3), r/r double hit lymphoma patients (7) and central nervous system lymphoma patients (8). In this particular patient group, ASCT or immunotherapeutic alone is thought to hardly enable long-term disease control. It is also worth mentioning that this novel combination strategy reduced the occurrence of severe cytokine release syndrome. Taken together, the combination of ASCT and CAR-T cell therapy has significant synergistic effects. Xiao et al. reviewed several CAR-T clinical trials that were shortly initiated after high-dose chemotherapy and ASCT. In addition, chemotherapy and radiotherapy are alternative bridging strategies, which synergize CAR-T cell function (9, 10).

## Combination with targeted therapy

The inadequate maintenance treatment after CAR-T cells infusion usually leads to therapy failure. CAR-T cells combined with small molecule inhibitors or immune checkpoint blockers may further improve the perseverance and function of CAR-T cells, reduce the tumor baseline burden, and create more favorable microenvironment for CAR-T cells, causing synergistic antitumor effects and improving the therapeutic efficacy.

Dual inhibition of histone deacetylase (HDAC) and Bruton’s tyrosine kinase (BTK) represents a promising combinatorial strategy. Zhu et al. presented a case of diffuse large B-cell lymphoma (DLBCL) with failed CD19 CAR-T cell therapy. Biopsy confirmed that the patient was CD19^+^ relapse. Next generation sequencing detected mutations in TP53 and NOTCH1. However, the patient achieved CR again after receiving chidamide and zanubrutinib. The author also discussed the possible mechanisms. For chidamide, it reverses CAR-T cell exhaustion through epigenetic regulation, inhibits mutant TP53 expression and up-regulates tumor antigen expression. For zanubrutinib, it reduces the expression of NOTCH1, inhibits AKT pathway, hinders the process of cell terminal differentiation, and increases memory CAR-T cell populations. Other epigenetic modulators, such as DNA methyltransferase inhibitors, decitabine and azacytidine, also have the potential of upregulating tumor antigen expression and reversing CAR-T cell exhaustion. Another second generation BTK inhibitor, acalabrutinib, was demonstrated to maintain the durability of CAR-T cells in several preclinical study. Thus, the superior combination drugs need to be urgently explored to achieve the optimal balance between efficacy, tolerability, and acceptability.

BTK inhibitors were also attempted in combination with BCL-2 inhibitors to further improve CAR-T cell efficacy. Nan et al. reported a patient with high-grade double-hit B-cell lymphoma, who had primary resistance to CD19 CAR-T cell therapy but achieved CR following management with ibrutinib and venetoclax. Ibrutinib not only has direct effect on B-cell lymphoma but also inhibits ITK, thus increasing T cell numbers and enhancing T cell function. Ibrutinib also promotes the release of cytokine and down-regulates the markers expression related to CAR-T cell exhaustion. Venetoclax induces the tumor antigen expression, enhances the persistence and anti-tumor efficacy of CAR-T cells, when combined with CAR-T cell therapy.

Immune checkpoint blockers PD-1/PD-L1 inhibitors can reverse T cell exhaustion and are considered to be an attractive strategy in combination use with CAR-T cells. Nan et al. reported a case of high-grade B-cell lymphoma, not otherwise specified that presented with primary resistance to CAR-T cell therapy without markedly elevated cytokine levels. The adding of pembrolizumab increased CAR-T concentrations and achieved clinical response over 10 months of duration. Hence, besides reversing T-cell exhaustion, pembrolizumab also promoted CAR-T cell activation and proliferation.

Lenalidomide can enhance CAR-T function and efficacy through multiple mechanism, including maintaining cell memory phenotype and increasing immune synapses. Li et al. reported a case of MYC/BCL2/BCL6 triple-hit lymphoma with TP53 mutation that achieved sustained CR for over two years after receving CD19 CAR-T cells infusoin and lenalidomide, suggesting a promising combination therapy.

PI3K-Akt-mTOR signaling is one of the most crucial oncogenic pathway networks in multiple cancers. However, the clinical efficacy of single-targeted agents against the PI3K-AKT-mTOR pathway is limited. Studies demonstrated that blocking this pathway promoted T cell epigenetic and metabolic reprogramming, thus facilitating the expansion and cytotoxicity of CAR-T cells. Hence, PI3K-Akt-mTOR inhibitors are popular combination therapy drugs and relevant clinical trials are underway to assess the effectiveness of different combination regimens prior to, concurrent with or after CAR-T cells. Other small molecule inhibitors, such as tyrosine kinase inhibitors, γ-secretase inhibitors and GSK-3βinhibitors, are promising candidates in maintenance therapy post immunotherapy.

## CAR-NK cell therapy

If patients are unable to tolerate CAR-T or developed resistance/relapse, CAR-NK cells may provide a safer, quicker, and more cost-effective way with no severe toxicities. Another unique advantage of CAR-NK immunotherapy is that the loss or downregulation of tumor target antigen didn’t significantly influence the function of CAR-NK cells. CAR-NK and allogenic NK cells constitute an ‘off-the-shelf’ immunotherapy that can be generated from healthy individuals and applied to different patients. Valeri et al. reviewed current strategies to overcome the resistance and improve the efficacy of CAR-NK therapy, including optimizing production protocols, eliminating T cell alloreactivity, surmounting replicative senescence, reversing the suppressive microenvironment, and restoring migration and homing into tumor bed. CRISPR/Cas9 genetic engineering is an exciting approach to optimize the CAR-NK cell function to eradicate refractory tumors. There are several phase I/II clinical studies under on right now to assess the effectiveness of CAR-NK therapy in treating hematological malignancies. 

## Concluding remarks

The reviews and original articles in this edition provide valuable insights for better understanding the strategies to optimize the infusion of CAR T-cell therapy in hematological malignancies. Dual-targeting CAR-T cells are popular ‘one-two-punch’ strategy to overcome CD19^-^ relapse. Novel CAR cell products are also quickly emerging, including universal ‘off-the-shelf’ CAR-T cells, next-generation CAR-T cells, and CAR-NK cells. CAR-T cell therapy was never an isolated treatment, and combinatorial strategies with traditional therapeutic systems or small molecules drugs are important future research directions. The key issues are who to engage or when, and how to engage best. Substantial ongoing studies are evaluating superior drugs and the optimal timing for combination therapies.

## Author contributions

TZ, TY and XY wrote the manuscript and finalized the figure; JW and WT reviewed and revised the manuscript and the figure. All authors contributed to the article and approved the submitted version.

## References

[1] Yang X Wei J . J Zhou. overcoming resistance to anti-CD19 CAR T-cell therapy in b-cell malignancies. Hematol Oncol (2022). doi: 10.1002/hon.3036 35635796

[2] Zhang Z Chen X Tian Y Li F Zhao X Liu J . Point mutation in CD19 facilitates immune escape of b cell lymphoma from CAR-T cell therapy. J Immunother Cancer (2020) 8(2). doi: 10.1136/jitc-2020-001150 PMC7539592 33023981

[3] Wei J Xiao M Mao Z Wang N Cao Y Xiao Y . Outcome of aggressive b-cell lymphoma with TP53 alterations administered with CAR T-cell cocktail alone or in combination with ASCT. Signal Transduct Target Ther (2022) 7(1):101. doi: 10.1038/s41392-022-00924-0 35399106 PMC8995369

[4] Wang N Hu X Cao W Li C Xiao Y Cao Y . Efficacy and safety of CAR19/22 T-cell cocktail therapy in patients with refractory/relapsed b-cell malignancies. Blood (2020) 135(1):17–27. doi: 10.1182/blood.2019000017 31697824

[5] Cordoba S Onuoha S Thomas S Pignataro DS Hough R Ghorashian S . CAR T cells with dual targeting of CD19 and CD22 in pediatric and young adult patients with relapsed or refractory b cell acute lymphoblastic leukemia: A phase 1 trial. Nat Med (2021) 27(10):1797–805. doi: 10.1038/s41591-021-01497-1 PMC8516648 34642489

[6] Tong C Zhang Y Liu Y Ji X Zhang W Guo Y . Optimized tandem CD19/CD20 CAR-engineered T cells in refractory/relapsed b-cell lymphoma. Blood (2020) 136(14):1632–44. doi: 10.1182/blood.2020005278 PMC7596761 32556247

[7] Wei J Mao Z Wang N Huang L Cao Y Sun W . Long-term outcomes of relapsed/refractory double-hit lymphoma (r/r DHL) treated with CD19/22 CAR T-cell cocktail therapy. Clin Transl Med (2020) 10(5):e176. doi: 10.1002/ctm2.176 32997409 PMC7507504

[8] Wu J Meng F Cao Y Zhang Y Zhu X Wang N . Sequential CD19/22 CAR T-cell immunotherapy following autologous stem cell transplantation for central nervous system lymphoma. Blood Cancer J (2021) 11(7):131. doi: 10.1038/s41408-021-00523-2 34267187 PMC8282870

[9] Perica K Flynn J Curran KJ Rivere I Wang X Senechal B . Impact of bridging chemotherapy on clinical outcome of CD19 CAR T therapy in adult acute lymphoblastic leukemia. Leukemia (2021) 35(11):3268–71. doi: 10.1038/s41375-021-01196-3 PMC8423852 33686196

[10] Pointer KB Pitroda SP Weichselbaum RR . Radiotherapy and immunotherapy: Open questions and future strategies. Trends Cancer (2022) 8(1):9–20. doi: 10.1016/j.trecan.2021.10.003 34740553

